# Neurogenic orthostatic hypotension: pathophysiology, evaluation, and management

**DOI:** 10.1007/s00415-012-6736-7

**Published:** 2012-11-20

**Authors:** Manuela Metzler, Susanne Duerr, Roberta Granata, Florian Krismer, David Robertson, Gregor K. Wenning

**Affiliations:** 1 Autonomic Function Laboratory, Division of Neurobiology, Department of Neurology, Innsbruck Medical University, Anichstrasse 35, Innsbruck, Austria; 2 Autonomic Dysfunction Center, AA3228 MCN, Vanderbilt University, Nashville, TN 37232-2195 USA

**Keywords:** Orthostatic hypotension, Neurogenic orthostatic hypotension, Parkinson's disease, Multiple system atrophy, Pure autonomic failure, Autonomic dysfunction

## Abstract

Neurogenic orthostatic hypotension is a distinctive and treatable sign of cardiovascular autonomic dysfunction. It is caused by failure of noradrenergic neurotransmission that is associated with a range of primary or secondary autonomic disorders, including pure autonomic failure, Parkinson’s disease with autonomic failure, multiple system atrophy as well as diabetic and nondiabetic autonomic neuropathies. Neurogenic orthostatic hypotension is commonly accompanied by autonomic dysregulation involving other organ systems such as the bowel and the bladder. In the present review, we provide an overview of the clinical presentation, pathophysiology, epidemiology, evaluation and management of neurogenic orthostatic hypotension focusing on neurodegenerative disorders.

## Introduction

According to consensus guidelines, orthostatic hypotension (OH) is defined as a sustained fall of systolic blood pressure by at least 20 mmHg or diastolic blood pressure by 10 mmHg within 3 min of standing or head-up tilt [16]. Since the magnitude of blood pressure drop also depends on baseline values, it was suggested that a drop of 30 mmHg may be a more appropriate criterion for OH in patients with supine hypertension [16]. Blood pressure is a clinical measure and the patients are not necessarily aware of its dysregulation The prevalence of OH increases with age and is commonly associated with neurodegenerative diseases including Parkinson’s disease (PD), dementia with Lewy bodies (DLB), multiple system atrophy (MSA) and pure autonomic failure (PAF). In the general aged population, the prevalence rates of OH range between 5 and 30 % [38, 47, 62, 76] (reviewed in [40]). A more extensive overview on the rate of occurrence is given in Table 1.

**Table 1:** Estimated prevalence of OH in different autonomic disorders

Condition	Prevalence rate (%)	References
Aging	10–30	[40]
Diabetes type I	8.4	[41]
Diabetes type II	7.4	[41]
Parkinson’s disease	37–58	[5, 66, 80]
Dementia with Lewy bodies	30–50	[4, 74, 75]
MSA	75	[34]
PAF	100	[2]

Modified according to [40] (with kind permission from Springer Science + Business Media B.V.)

Hallmark symptoms upon postural challenge include dizziness, visual disturbances, presyncope and syncope [25, 52]. However, the majority of patients experience more subtle general complaints, such as tiredness, impaired cognitive performance [57], weakness, fatigue, leg buckling, visual blurring and orthostatic dyspnea [43]. Patients may also experience discomfort in the head, neck, shoulders or the chest. The latter may be reminiscent of anginal pain in the absence of coronary heart disease [60]. Symptoms are usually aggravated during hot weather or fever, after heavy meals, during prolonged standing and early in the morning [44]. In many patients, the worsening of symptoms early in the morning is caused by nocturnal diuresis due to the increase in supine blood pressure as shown in a study involving MSA patients [55].

## Pathophysiology

Consciousness is critically dependent on continuous cerebral blood flow, and is lost within 6 s of shutdown of cerebral blood flow in human subjects [73]. Thus any stimulus or condition that perturbs cerebral perfusion may cause symptoms. Gravitationally mediated pooling of venous blood in the lower half of the body (i.e. legs and abdomen) begins almost immediately upon postural challenge and most of the venous pooling takes place within the first 10 s [10, 72]. The amount of blood transferred to lower body parts depends on the type of orthostatic stress and is estimated to 500–1,000 ml [65, 71, 72]. In addition, it was shown that plasma volume decreases during orthostatic stress [54]. As a consequence, the venous return to the heart is reduced which leads to a reduction in stroke volume by affecting end-diastolic filling of the right atrium (“Frank–Starling” relationship, [37]) resulting in a 20 % decrease in cardiac output [77]. The compensatory reflex response is mainly mediated by the baroreceptors (arterial mechanoreceptors) which cause increased sympathetic outflow and suppressed vagal nerve activity resulting in increased peripheral resistance and improved venous return ultimately yielding to increased cardiac output [72]. However, in OH patients, an impaired increase in peripheral resistance could be observed that is most likely caused by disturbed neural reflex vasoconstriction [82].

It is noteworthy that two distinct kinds of pathological processes can dramatically alter autonomic blood pressure regulation in human subjects: baroreflex failure (BF) and neurogenic orthostatic hypotension (NOH). In BF, there is loss of afferent baroreflex engagement of central mechanisms of blood pressure control. However, central stimuli (such as anxiety, pain, anger, or excitement) can still engage an otherwise functional peripheral sympathetic system. These BF patients have extreme surges of blood pressure largely dependent on emotional state. Some of these surges (elevations above 250 mmHg have been observed) are among the highest blood pressures encountered in contemporary clinical medicine [27, 33, 59]. Blood pressure may, however, be normal or occasionally low in the BF subjects when they are tired, during rest, or when they are sedated. Posture plays a relatively small role in the blood pressure level in many of these patients, although it has been reported in rare cases of BF with medullary lesions [12]. BF is generally caused by bilateral structural lesions in the carotid sinuses, the glossopharyngeal nerves or brainstem due to tumor, injury, or other damage to afferent pathways.

In contrast to the afferent or central lesion of BF, there is failure of noradrenaline release from sympathetic vasomotor neurons in NOH [16]. Loss of homeostatic mechanisms to control blood pressure fluctuations may contribute to the supine hypertension (systolic pressure >180 mmHg and/or diastolic pressure >110 mmHg) commonly encountered in the spectrum of NOH [20]. This has to be considered when treating NOH in order to avoid the risk of chronic high blood pressure on the one hand and the risk of falling with its secondary consequences on the other hand.

## Predisposing factors

Orthostatic hypotension is influenced by a range of factors; cross-sectional analysis not only suggests an influence of age, but also drug effects and orthostatic stress in neurological disorders, particularly PD, DLB and MSA as well as autonomic neuropathies [5, 40, 49, 66, 80]. The association between OH and advanced age may be explained by a number of predisposing factors that occur along with aging, including changes in baroreflex function, inadequate vasoconstrictor responses, reduced cardiac and vascular compliance, reduced blood volume and impaired efficiency of the skeletal muscle pump [16]. In addition, dehydration, deconditioning and poor nutrition contribute to the development of OH in the elderly population [58].

Another factor massively influencing OH prevalence is the effect of medication. Elderly subjects commonly require medications altering blood pressure, such as diuretics or antihypertensives, which are well known to either cause or exacerbate OH. In addition, alpha-adrenoceptor antagonists in the treatment of benign prostatic hyperplasia, tricyclic antidepressants, vasodilatators, sympatholytics, and antiparkinsonian agents can increase risk of OH by impairing sympathetic tone or reducing peripheral vascular resistance [45]. Further, increased orthostatic stress in patients with parkinsonian conditions may be observed early in the morning, with a rise in core temperature, in activities which increase intrathoracic pressure (e.g. defecation, coughing) [64], prolonged standing, exertion, alcohol or carbohydrate ingestion [61]. A structured list of OH causes is presented in Table 2.

**Table 2:** Causes of orthostatic hypotension

Autonomic disorders without CNS or PNS involvement
Pure autonomic failure (PAF)
Autonomic disorders with brain involvement
Multiple system atrophy (MSA)
Wernicke Korsakoff syndrome
Posterior fossa tumors
Baroreflex failure
Olivopontocerebellar atrophy
Dementia with Lewy bodies
Adult-onset autosomal dominant leukodystrophy (ADLD)
Autonomic disorders with spinal cord involvement
Traumatic tetraplegia
Syringomyelia
Subacute combined degeneration
Multiple sclerosis
Spinal cord tumors
Autonomic neuropathies
The acute autonomic neuropathies
Autoimmune autonomic ganglionopathy (AAG; acute pandysautonomia)
Acute paraneoplastic autonomic neuropathy
Guillain–Barre syndrome
Botulism
Porphyria
Drug induced acute autonomic neuropathies
Toxic acute autonomic neuropathies
The chronic peripheral autonomic neuropathies
Pure adrenergic neuropathy
Combined sympathetic and parasympathetic failure (autonomic dysfunction clinically important)
Amyloid
Diabetic autonomic neuropathy
Paraneoplastic autonomic including panautonomic neuropathy
Sensory neuronopathy with autonomic failure (most commonly associated with Sjogren’s syndrome)
Familial dysautonomia (Riley-Day syndrome)
Autoimmune autonomic neuropathy
Dysautonomia of old age

Modified according to [40], Table 3 (with kind permission from Springer Science + Business Media B.V.)

## NOH in neurodegenerative disease

Neurogenic orthostatic hypotension can arise from primary neurodegenerative disorders or can be secondary to systemic conditions that influence peripheral nerve function [22]. PD, DLB, MSA and PAF belong to a category of neurodegenerative disorders known as α-synucleinopathies due to their cellular hallmark feature that is α-synuclein inclusion pathology [46]. The prevalence of NOH in PD ranges from 16 to 58 % [66, 80]. Likewise, in DLB symptomatic OH is found in 30–50 % of the patients separating DLB from other dementias including Alzheimer’s disease and frontotemporal dementia [4, 74, 75]. Both PD and DLB show markedly decreased myocardial [123I]-metaiodobenzylguanidine uptake indicating severe impairment of the cardiac sympathetic innervations [7, 24]. MSA-associated NOH symptoms are present in more than two-thirds of all patients [34] and were, therefore, included into consensus diagnostic criteria [18]. PAF is a disease which is characterized by severe NOH associated with insidious onset, slow progression, modest gastrointestinal impairment, marked supine hypertension and often very low plasma noradrenalin levels representing a characteristic prototype of NOH [30].

## Evaluation

Patients with NOH may split into two groups according to the site of the lesion with (1) disturbed central autonomic pathways and intact peripheral noradrenergic innervation or (2) loss of peripheral noradrenergic fibers [22]. Disruption of central autonomic pathways is commonly associated with normal or only slightly reduced plasma norepinephrine concentrations whereas the second group is characterized by low norepinephrine levels [19, 21].

**Fig. 1:**
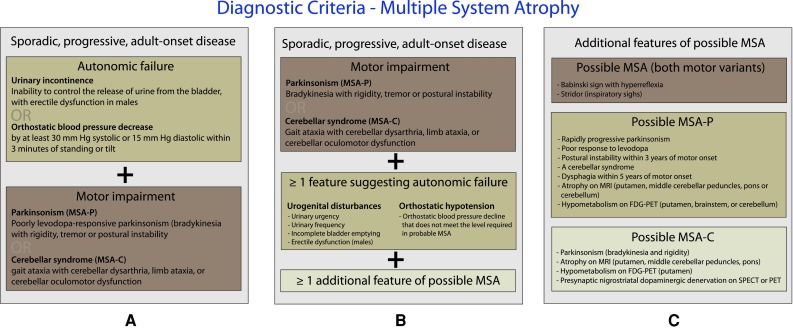
Consensus criteria for the diagnosis of MSA. Modified according to [18]. **a** Diagnostic criteria for the diagnosis of probable MSA. **b** Diagnostic criteria for the diagnosis of possible MSA. **c** Additional features suggestive of MSA required for a diagnosis of possible MSA

The first step in the work-up of patients presenting with symptoms suggestive of NOH is the exclusion of potentially harmful causes such as acute bleeding and dehydration. Next, non-neurogenic causes (reviewed in [14]) including drugs, reduced cardiac output, endocrine disorders and excessive vasodilatation should be considered. In the absence of apparent causes, further work-up by cardiac autonomic function testing (CAFT) is indicated. Blood pressure and heart rate should be recorded in supine position and after 3 min of standing [16, 35]. In addition, Holter monitoring and 24-h blood pressure profiles accompanied by an accurate diary may be useful to determine the effects of daily life stimuli [48]. Moreover, the parasympathetic nervous system could be distinguished from the sympathetic adrenergic system by functional assessments. Heart rate variability upon deep respiration and during a Valsalva maneuver target the parasympathetic nervous system whereas blood pressure responses upon head-up tilt and during Valsalva maneuver point towards the sympathetic system [1, 11, 50]. Actions known to raise the blood pressure including isometric exercise, the cold pressor test (immersing the hand in ice slush for 90 s) and mental arithmetic may be used to examine activation of different afferent or central pathways [48, 50]. Moreover, a careful work-up is required to diagnose neurological disorders underlying NOH. Present diagnostic criteria for PD are listed in Table 3 and consensus criteria for the diagnosis of MSA are presented in Fig. 1. Finally, the diagnosis of diabetic neuropathy requires utilization of clinical and physiological measures [3].

**Table 3:** Queen Square Brain Bank clinical diagnostic criteria for the diagnosis of Parkinson’s disease

Step 1. Diagnosis of parkinsonian syndrome
Bradikinesia (slowness of initiation of voluntary movement with progressive reduction in speed and amplitude or repetitive actions)
And at least one of the following:
Muscular rigidity
4–6 Hz rest tremor
Postural instability not caused by primary visual, vestibular, cerebellar, or proprioceptive dysfunction
Step 2. Exclusion criteria for Parkinson’s disease
History of repeated strokes with stepwise progression of parkinsonian features
History of repeated head injury
History of definite encephalitis
Oculogyric crises
Neuroleptic treatment at onset of symptoms
More than one affected relative
Sustained remission
Strictly unilateral features after 3 years
Supranuclear gaze palsy
Cerebellar signs
Early severe autonomic involvement
Early severe dementia with disturbances of memory, language, and praxis
Babinski signs
Presence of a cerebral tumor or communicating hydrocephalus on CT scan
Negative response to large doses of l-dopa (if malabsorption excluded)
MPTP exposure
Step 3. Supportive prospective positive criteria of Parkinson’s disease. Three or more required for diagnosis of definite Parkinson’s disease:
Unilateral onset
Rest tremor present
Progressive disorder
Persistent asymmetry affecting the side of onset most
Excellent response (70–100 %) to l-dopa
Severe l-dopa-induced chorea
l-dopa response for 5 years or more
Clinical course of 10 years or more
Hyposmia
Visual hallucination

Reprinted from [36] Copyright (2009), with permission from Elsevier

### Case presentations

#### Case I

A 77-year-old female was admitted to the hospital after a transient loss of consciousness. At the hospital, she indicated that she did not perceive the present “attack” and quickly felt comfortable again. Moreover, she was not aware of any dizziness, visual disturbances or light-headedness prior to her fainting spell. A history of recurring syncope that began in childhood was noted previously. Of note, the patient reported several cardiac risk factors including hyperlipidaemia, arterial hypertension and type 2 diabetes. Third-party descriptions did not suggest epileptic-like convulsions. During inpatient stay, another fainting fit was observed and measurements indicated low blood pressure. Subsequently, Holter monitoring and 24-h blood pressure profiles remained within normal range. Arterial hypertension was classified as non-dipper with nocturnal blood pressure drop of 3.8 % systolic and 4.9 % diastolic.

Tilt-table testing elicited a marked NOH with a supine blood pressure of 112/61 mmHg dropping to 68/46 mmHg after 3 min of head-up tilt, despite a constant heart rate of 62 bpm. A tilt-induced syncope occurred after 3.5 min. Video monitoring observed orofacial automatisms and dystonic-myoclonic movements of the upper limbs as manifestations of cerebral hypoperfusion.

In the present case, medical history and clinical examinations suggest severe NOH in the context of cardiovascular autonomic diabetic neuropathy.

#### Case II

A 67-year-old male reported a 2-year history of progressive gait unsteadiness which initially started with slight balance difficulties. More recently, slurred speech, recurring falls without serious injuries and impaired fine motor skills appeared. In addition, the patient described symptoms suggestive of presyncope. In the clinical examination a cerebellar syndrome accompanied by mild akinetic-rigid parkinsonism was observed. Cerebral magnetic resonance imaging detected pontine and cerebellar atrophy. Presyncopal symptoms were further investigated by a simple standing test which confirmed the suspected diagnosis of OH. Within 3 min of head-up tilt in tilt-table testing, a blood pressure drop of 76 mmHg systolic and 51 mmHg diastolic associated with an inadequate increase of 8 bpm in heart rate appeared and further underscored the diagnosis of NOH. Overall, the patient met the Gilman criteria of probable MSA and received 9-α-fluorohydrocortison which alleviated OH symptoms substantially as well as levodopa–benserazide which mediated a modest benefit towards parkinsonian symptoms only.

## Management

A structured approach is important in the management of patients with NOH. Wherever possible, underlying causes should be identified by thorough work-up and the treatment strategy adapted accordingly. In addition, the magnitude of symptoms and the presence of asymptomatic OH should be considered. Available treatment options range from non-pharmacological options to aggressive drug therapy. While therapy of non-neurogenic OH is mostly straight-forward, NOH is often difficult to treat and a combination of non-pharmacological measures and drugs is required. Pharmacological agents can lead to different responses in patients with central neurodegeneration compared to those with peripheral neurodegeneration, and the latter has to be considered in the treatment as well.

Nevertheless, NOH massively affect patients’ quality of life because of the disabling symptoms of autonomic failure. However, most of these symptoms could be alleviated by non-pharmacological and pharmacological measures. Therapeutic interventions should be implemented stepwise depending on the severity of symptoms. If non-pharmacological measures do not attenuate NOH symptoms sufficiently, pharmacological interventions may become necessary. Nevertheless, supine hypertension has to be taken into consideration in pharmacological treatment [15].

### Non-pharmacological interventions

Non-pharmacological measures should be considered first in NOH. Such measures include a stepwise raising from supine to standing position, physical exercise in order to avoid deconditioning, taking care of proper defection and compression stockings [14]. An abdominal bandage may also be useful in attenuating orthostatic dysregulation by restricting splanchnic blood pooling [9] and, similarly, physical maneuvers such as night time head-up tilt, leg-crossing, thigh contraction and squatting improve cerebral perfusion [79]. The spreading of total daily carbohydrate intake to multiple smaller meals was shown to beneficially affect orthostatic symptoms [39]. The effect of 500 ml oral water ingestion typically increases blood pressure 20–30 mmHg for about an hour, and sometimes greatly potentiates the pressor effect of other drugs [28]. Finally, adequate salt and fluid intake may be useful with dietary sodium intake of at least 10 g per day and a fluid intake of more than 2 l per day [14, 29, 68, 78]. However, it has to be considered that increased fluid and salt intake may be harmful in patients with concomitant renal dysfunction and, thus, dietary fluid and salt intake requires regular check-up.

### Pharmacological treatment

Two different mechanistic targets are approached in the pharmacological treatment of NOH, namely volume expansion and vasoconstriction. In patients failing to respond appropriately to high salt diet and increased fluid intake, the prescription of 9-α-fluorohydrocortison, a synthetic mineralocorticoid, is indicated in order to increase plasma volume by renal sodium retention. Intriguingly, both of the latter effects return to normal over time, suggesting that increased peripheral vascular resistance (PVR) contributes to the observed pressor effect [8]. At the same time, PVR is the limiting factor of 9-α-fluorohydrocortison treatment resulting in dose-dependent supine hypertension [8]. Other adverse events include ankle edema, hypokalemia, headache and congestive heart failure.

On rare occasions, the vasopressin-analogue desmopressin could be applied to reduce nocturnal diuresis and expand plasma volume [51, 63]; however, those patients with impaired release of vasopressin due to neurodegeneration in hypothalamic areas such as MSA patients benefit the most [31]. Nevertheless, side effects including intoxication and hyponatremia have to be considered [51].

Bearing in mind that impaired norepinephrine release from sympathetic neurons is the central mechanism in NOH pathophysiology, sympathomimetic drugs yielding to vasoconstriction may be helpful in the treatment of NOH, particularly in patients where plasma volume increase was insufficient to abolish orthostatic symptoms. However, so far, the only drug which has been approved by regulatory authorities (i.e. FDA, EMEA) for the treatment of NOH is the peripheral and directly acting α_1_-adrenoreceptor agonist midodrine. In two multi-centre double-blind placebo-controlled studies midodrine mediated beneficial effects that ameliorated orthostatic symptoms and increased standing blood pressure [42, 81]. More recently, the norepinephrine precursor l-dihydroxyphenylserine (l-DOPS, droxidopa) was shown to be effective in NOH-associated neurodegenerative conditions [32, 53], and this agent seems near FDA approval for NOH in the United States. In rare causes of NOH like dopamine-beta hydroxylase deficiency, where noradrenaline is absent because of lack of the functional enzyme which produces noradrenaline, droxidopa can occasionally elicit a “Lazarus effect”. Individuals with lifelong severe orthostatic hypotension and inability to stand for more than 2 min without losing consciousness may improve with droxidopa treatment to such an extent that may enable patients to successfully complete a marathon run [17]. Other sympathomimetics, particularly those with mixed or indirect effects, were either inferior to midodrine [13] or were not studied systematically [35]. However, these drugs may still be helpful in individual cases in which the patient did not respond to common pharmacologic options. A beneficial effect has been reported for other drugs in patients who have had limited or no response to the previously mentioned therapies. Of note, someone has to be well aware of the fact that all of the following drugs have to be administered off-label. Treatment of normocytic, normochromic anaemia in patients using erythropoietin increased standing blood pressure and improved orthostatic intolerance [6, 26, 56]. The cholinesterase inhibitor pyridostigmine improved ganglionic transmission and vascular adrenergic tone in primarily upright position, mediating a slight increase in diastolic blood pressure during standing without worsening supine hypertension [70]. Another drug being tested was yohimbine, which is known to release noradrenaline from sympathetic nerves via increasing neuronal output and antagonizing α_2_-adrenoceptors [23]. Intriguingly, patients with intact noradrenergic innervation experienced substantial increases in blood pressure and plasma noradrenaline levels, whereas attenuated effects were observed in patients with noradrenergic denervation [67, 69].

## Conclusion

Neurogenic orthostatic hypotension can seriously impair patients’ quality of life and is associated with increased morbidity, especially in the elderly. In several neurological diseases associated with autonomic failure, NOH is a major contributor to disease burden and reduced quality of life. A structured approach is important in the management of patients with NOH. Non-pharmacological interventions should be the first line of therapy. If the symptoms persist and the patients are severely affected, pharmacological interventions are required.
